# Atomic and electronic structure of Lomer dislocations at CdTe bicrystal interface

**DOI:** 10.1038/srep27009

**Published:** 2016-06-03

**Authors:** Ce Sun, Tadas Paulauskas, Fatih G. Sen, Guoda Lian, Jinguo Wang, Christopher Buurma, Maria K. Y. Chan, Robert F. Klie, Moon J. Kim

**Affiliations:** 1 Department of Materials Science and Engineering, the University of Texas at Dallas, Richardson, TX 75080, USA; 2 Department of Physics, University of Illinois at Chicago, Chicago, IL 60607, USA; 3 Center for Nanoscale Materials, Argonne National Laboratory, Lemont, IL 60439, USA

## Abstract

Extended defects are of considerable importance in determining the electronic properties of semiconductors, especially in photovoltaics (PVs), due to their effects on electron-hole recombination. We employ model systems to study the effects of dislocations in CdTe by constructing grain boundaries using wafer bonding. Atomic-resolution scanning transmission electron microscopy (STEM) of a [1–10]/(110) 4.8° tilt grain boundary reveals that the interface is composed of three distinct types of Lomer dislocations. Geometrical phase analysis is used to map strain fields, while STEM and density functional theory (DFT) modeling determine the atomic structure at the interface. The electronic structure of the dislocation cores calculated using DFT shows significant mid-gap states and different charge-channeling tendencies. Cl-doping is shown to reduce the midgap states, while maintaining the charge separation effects. This report offers novel avenues for exploring grain boundary effects in CdTe-based solar cells by fabricating controlled bicrystal interfaces and systematic atomic-scale analysis.

Polycrystalline zinc blende CdTe is used as an absorber layer in thin film CdTe/CdS photovoltaic (PV) devices. The heterojunction diode has drawn significant attention due to its outstanding solar energy efficiency-to-cost ratio, and is the current leader in the thin film PV market^1^. The best laboratory energy conversion efficiency of CdTe PV devices now reaches 21.5%, but there is still a considerable gap to the Shockley-Queisser limit of ~32%^2^,^3^,^4^. One of the main factors limiting the conversion efficiency of poly-CdTe solar cells is low minority carrier lifetime, 1–10 ns, which is attributed to non-radiative recombination at grain boundaries (GBs), dislocations, and point defects^4^. The importance of understanding the atomic structure and electronic behavior of such defects in elemental and compound semiconductors has long been recognized by the PV community^5^. More specifically, GBs and dislocations are generally viewed as detrimental to thin film PV device applications since they tend to introduce deep defect levels in the band-gap which act as effective recombination centers for photo-generated carriers, paths for forward current, or scattering centers for free carriers^6^. However, the role of GBs and dislocations in limiting the device performance is still not fully understood since, for example, polycrystalline CdTe and Cu(In,Ga)Se_2_ (CIGS) PV devices outperform their single-crystalline PV counterparts, which achieve conversion efficiency of less than 10%^7^. In particular, poly-CdTe devices have short-circuit currents, *J*_sc_, and fill factors that are significantly higher than single-crystalline CdTe. One hypothesis is that local band-bending established at GBs and dislocations can effectively separate electrons and holes, reducing the charge carriers’ recombination, and thus increasing current collection of the polycrystalline device^5^,^8^. A common approach for describing the effect a dislocation has on the electronic structure is to consider two regions^9^: (i) the core region where bonds are broken and reconstructed, and the local atomic environments deviate significantly from the bulk crystalline structure, (ii) the extended strain field around the core forming the so-called ‘Cottrell atmosphere’^10^,^11^.

Despite the importance of GBs and dislocations in semiconductor device applications, the study of these defects had been hindered by difficulties in obtaining the atomic and electronic structure of individual dislocation cores in polycrystalline devices, and separating the effects of a single grain boundary from the overall transport properties of the device. Determination of the atomic and electronic structure of grain boundaries requires an approach combining high spatial resolution imaging with high energy resolution spectroscopy and atomistic simulations^11^,^12^. Using aberration-corrected scanning transmission electron microscopes (AC-STEM), we are able to obtain atomic-level information from dislocation cores and compare it with first-principles modeling predictions^13^,^14^,^15^,^16^.

To date, theoretical and experimental studies of CdTe GBs have covered only tilt Σ3 (111), and Σ9 (112) interfaces, as found within polycrystalline CdTe crystals^8^,^17^,^18^,^19^,^20^,^21^,^22^. Such an approach of studying GB atomic structures in poly-crystalline thin films is dependent upon luck of being able to find two grains with exactly aligned and viewable zone axis for STEM imaging. A better approach would be to fabricate specific grain boundaries with well-defined misorientation angles using two bulk CdTe wafers. Here, we have developed a method for bonding two single crystal samples together into one bicrystal, the so called wafer bonding method, which is used to create artificial GBs with well-defined misorientation angles, and interfacial planes, thereby allowing us to explore a large portion of the grain boundaries’ configuration space^23^,^24^,^25^,^26^. The wafer bonding technique has been used to investigate interfaces in MgO, Bi_2_Te, and Al_2_O_3_^11^,^27^,^28^,^29^.

In this paper, we have combined high-angle annular dark-field (HAADF) imaging in an AC-STEM, first-principles density functional theory (DFT) modeling, and geometrical phase analysis (GPA), to study a [1–10]/(110) 4.8° tilt GB created by wafer boding. We find that the grain boundary is composed of several different types of Lomer dislocations, and determine the atomic structures of the dislocation cores, which provide evidence of the reaction mechanism of these dislocations. The electronic structure, computed using first-principles DFT on realistic, STEM-matched, models containing up to 1917 atoms, shows significant creation of mid-gap states and distinct charge-channeling tendencies at the dislocation cores. Using the precise atomistic model obtained from STEM imaging on the created artificial grain boundary, the effects of Cl passivants on the mid-gap states and charge-channeling effects are shown with DFT calculations, demonstrating the effectiveness of the current approach towards understanding and improving grain boundaries in semiconductor electronics.

## Results

### Validation of dislocation array in bicrystal

**Figure 1:**
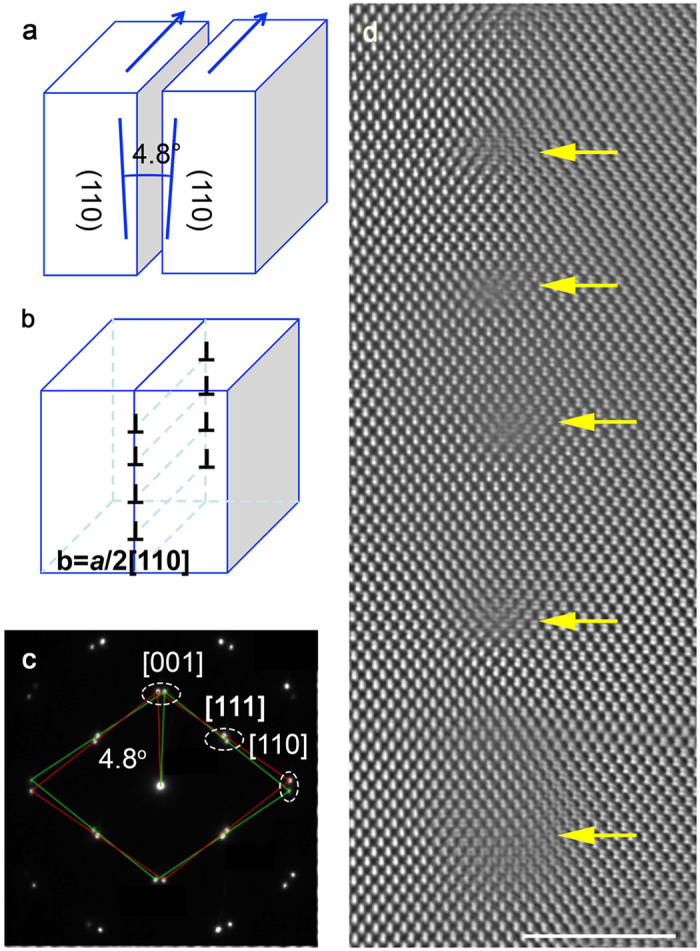
Verification of dislocation array. (**a**) CdTe bicrystal with [1–10]/(110) 4.8° tilt grain boundary. (**b**) Dislocation array expected for the bicrystal consisting of (*a*/2)[110] edge dislocations at ~5.5 nm intervals. (**c**) SADP taken at the boundary. (**d**) A representative cross-sectional HAADF-STEM image of the boundary viewed along [1–10] direction. Scale bar, 5 nm.

A [1–10]/(110) 4.8° CdTe tilt GB was fabricated by wafer bonding. The symmetrical *θ* = 4.8° tilt angle was achieved by cutting two single crystal CdTe crystals by 2.4° from the (110) plane as shown in Fig. 1a,b. Selected area diffraction pattern (SAPD) taken from the bonded interface confirm the misorientation angle of the grain boundary using the splitting of the Bragg spots shown in Fig. 1c. Figure 1d shows an atomic-resolution HAADF image of the [1–10]/(110) 4.8° CdTe tilt GB. In order to accommodate a tilt angle *θ,* the interface is expected to contain a periodic array of evenly spaced dislocations having on average |**b**| edge components. The dislocation spacing, *D,* is determined by Frank’s formula *D* = |**b**|/*θ*, where |**b**| is the magnitude of the dislocation’s Burgers vector. Using atomic-resolution HAADF images, we find that the average spacing between two adjacent dislocations is 5.6 ± 1.0 nm. This is consistent with *D* = 5.5 nm, as expected from Frank’s formula for a 4.8° tilt grain boundary with a dislocation’s Burgers vector of **b** = (*a*/2)[110]. Such a Burgers vector indicates the pure edge character of the dislocations at the grain boundary. In addition, we did not find any significant interfacial faceting or interfacial amorphous layers, demonstrating that our bicrystal fabrication has been successful.

### Multiple configurations of Lomer dislocations

**Figure 2:**
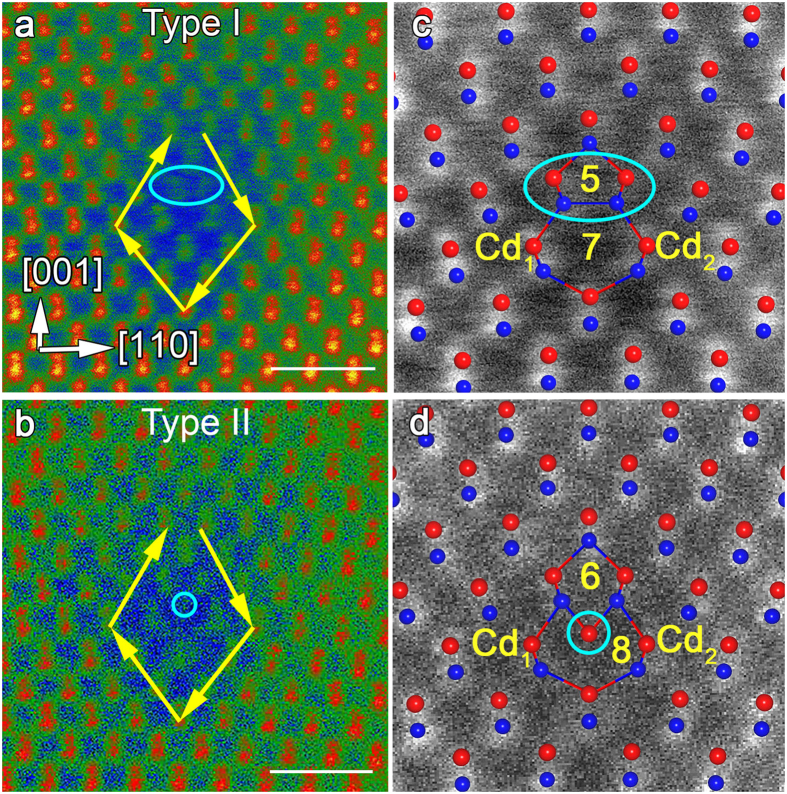
Atomic structure of Type I and II dislocation cores. HAADF-STEM images of Lomer dislocations viewed from [1–10] direction. (**a**) Type I dislocation with relevant crystallographic directions and (**c**) corresponding DFT relaxed atomic structure overlayed on the HAADF image. (**b**) HAADF image of Type II dislocation and (**d**) corresponding DFT relaxed atomic structure overlayed on the image. Burgers circuits surrounding the dislocation cores in (**a**,**b**) marked by yellow arrows indicated that the Burgers vector of the Type I and II dislocations are (*a*/2)[110]. Scale bar, 1 nm.

**Figure 3:**
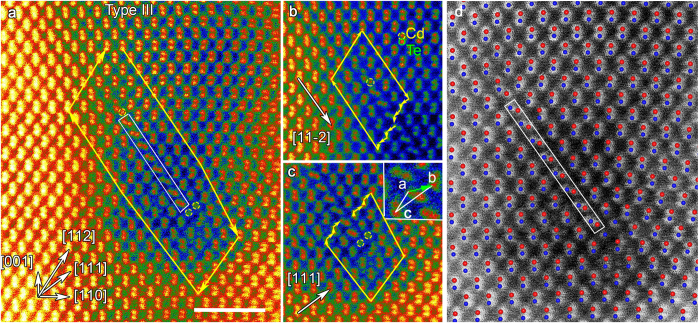
Atomic structure of Type III dislocation (**a**) HAADF-STEM image of the Type III dissociated dislocation. The intrinsic stacking fault is bounded by two partial dislocations. The Burgers circuit surrounding the whole configuration indicates that the Burgers vector of the dislocation (*a*/2)[110], same as Type I and II Lomer dislocations. Two ‘extra’ {111} half-planes can be seen squeezed-in parallel to the stacking fault and terminated at the lower core. Scale bar is 2 nm. (**b**,**c**) HAADF images of the upper and lower partial dislocation cores shown separately. The Burgers vector of each dislocation is identified as (**b**) **b**_**1**_ = (*a*/6)[2−1−1], which is Cd-terminated 30° Shockley partial, and (**c**) **b**_**2**_ = (*a*/6)[141]. (**d**) DFT-relaxed atomic structure of Type III instrinsic stacking fault overlayed on the HAADF-STEM image.

The great majority of dislocations found along the grain boundary have a Burgers vector **b** = (*a*/2)[110]. This type of dislocations can also be found in materials with diamond cubic crystal structure (Si, diamond), and is called a Lomer dislocation. These pure-edge dislocations are immobile since their slip plane would take place on {001} planes, which do not belong to the usual <110 > {111} slip system of CdTe. Three distinct types of Lomer dislocations can be found in our HAADF-STEM images, which we will call Type I, II and III throughout the remainder of this discussion. Burgers circuits around the different types of dislocations are shown in Figs 2a,b and 3a, respectively. The DFT-relaxed atomic structures of Type I, II and III dislocations are shown overlaid on experimental HAADF-STEM images in Figs 2c,d and 3d, respectively, demonstrating that the DFT-relaxed atomic structures match elegantly the STEM images. The atomistic model construction procedures and DFT calculations are described in the Methods Section and Supplementary Fig. 1. Although all the Lomer dislocation types have the same total Burgers vector, **b**, the atomic structures are quite different. In the Type I configuration, shown in Fig. 2a,c, the dislocation core structure consists of five- and seven-fold ring units, with two dumbbell pairs terminating the core. This type of coordination has been predicted for the edge dislocations in diamond cubic materials by Hornstra^30^ and has been observed in classical simulations by Nandedkar and Narayan^31^. Type II dislocations contain eight-fold ring, as shown in Fig. 2b,d. The main difference between Type I and II is that the core in Type II contains an additional single atomic column between Cd_1_ and Cd_2_. The line profiles along Cd_1_–Cd_2_ direction of Type I in Supplementary Fig. 2a–c shows that the distance between Cd_1_ and Cd_2_ is about 0.65 nm. The line profiles for Type II dislocations (Supplementary Fig. 2d–f) reveals the extra column between Cd_1_ and Cd_2_, and the distance between the two Cd columns increases to around 0.72 nm. Based on the surrounding columns and DFT structural calculations, discussed later, we identify the extra column as Cd. The structure of Type III is given in Supplementary Fig. 3.

These Lomer dislocations can be seen as two 60° elemental dislocations that have merged from different {111} planes. The intersection angle is acute and the reaction can be written as:


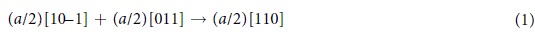


One interpretation of the difference between Type I and II cores is that the intersection may not happen at the vertex defined by the two {111} planes on which dislocations glide, but they may slip past each other before being ‘locked-in’ by strain fields. Future molecular dynamics calculations may help shed further light on this reaction.

An example of a type III dislocation, which consists of two cores separated by an intrinsic stacking fault, is shown in Fig. 3a. From the Burgers circuit surrounding the entire configuration, the Burgers vector is identified as **b** = (*a*/2)[110] (Fig. 3a). Images of the upper and lower dislocation cores, along with Burgers circuits, are shown separately in Fig. 3b,c. The projected component of the Burgers vector in Fig. 3b is (*a*/12)[11−2]. This can readily be identified as a glide set Shockley 30° partial dislocation, with a corresponding Burgers vector of **b**_**30**°_ = (*a*/6)[2−1−1]. The core structure of the 30° partial dislocation consists of a single Cd atomic column. Earlier reports on the atomic and electronic structures of Shockley dislocations in CdTe have shown that the Cd dislocations act as shallow donors while the Te cores act as shallow acceptors, as indicated by the Fermi levels in the band structure diagrams^5^. Unlike the upper 30° partial dislocation, in the lower dislocation core in Fig. 3c, it is not readily apparent in which crystallographic direction the projected Burgers vector is pointing. In order to find the Burgers vector, we take the total Burgers vector **b** from Fig. 3a and subtract from it the 30° partial according to equation (2):


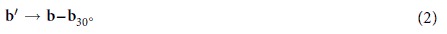


The Burgers vector of the lower core is therefore **b′** = (*a*/6)[141]. According to previous studies in other materials, the unusual (a/6) <411> partial dislocation can be seen as the reaction of a perfect 60° (a/2) <110> and 90° (*a*/6) <211> partial dislocation^32^,^33^.

As shown in equation (1), Lomer dislocations can be interpreted as two merged 60° elemental dislocations, and can be visualized using Thompsons tetrahedron notation (Supplementary Fig. 5):


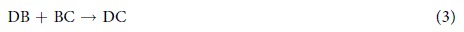


In order to understand how Type III dislocation formed, we may consider two probable scenarios: i) the dissociation of a previously perfect Lomer dislocation (Supplementary Fig. 5) or ii) the reaction of a 60° dislocation with an already dissociated 60° dislocation. Frank’s **b**^**2**^ criteria^34^, which compares elastic energy states of dislocations prior and post reaction, tells us that it is energetically more favorable for the latter process to take place. In fact, it can be seen that the undissociated Lomer dislocation has lower elastic energy compared to the final product after dissociation into two partials and a stacking fault. However, the elastic energy is reduced when two isolated dislocations, one of which is already dissociated, merge. In the following we will assume the latter scenario and discuss the reaction in more details.

The dissociation of a 60° dislocation on the (111) plane can be written as:


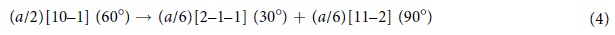


or using the Thomson tetrahedron notation, as:


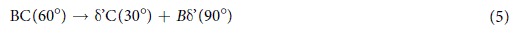


An intrinsic stacking fault is created in this step and the 30° partial is placed at the upper core of Fig. 3a. The second 60° dislocation reacts with the 90° partial (*a*/6)[11−2] or Bδ’ in Supplementary Fig. 4 to form the partial dislocation at the lower core of Fig. 3c, described as:


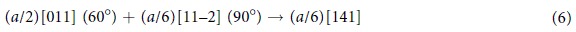


The dislocation reactions of the whole Type III dislocation are, therefore, given as:


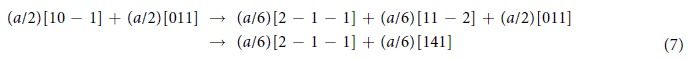


The two partial dislocations can be seen to possess screw components of equal magnitude but opposite direction, such that the whole configuration is still of pure-edge character. The distance between the partial dislocations is on average ~4.0 nm. The dissociation can take place on either one of the two distinct {111} planes, as seen in our STEM imaging analysis. However, we have not observed Lomer dislocations dissociated on both planes simultaneously. We note that, to our knowledge, this type of dislocation has not been reported in CdTe before.

### Geometrical phase analysis (GPA) to determine strain field

**Figure 4:**
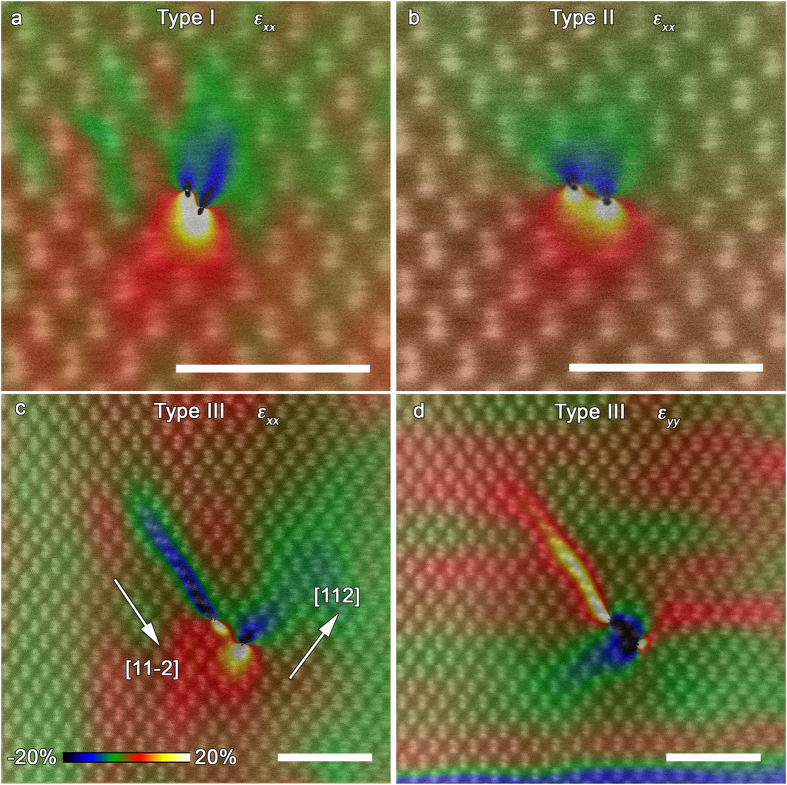
Strain field distribution of the dislocation cores. Strain maps of the in-plane *ε*_*xx*_ calculated by the GPA for the type I (**a**), II (**b**) and III (**c**) dislocations, and *ε*_*yy*_ for Type III (**d**) dislocation. The HAADF-STEM images are overlapped on the strain maps to indicate the exact location of the dislocation cores. The color bar indicates change in strain intensity from −20% (compressive) to 20% (tensile). Scale bar corresponds to 2 nm.

To quantify the local structural differences of the different types of Lomer dislocations, we perform a GPA of the experimental HAADF-STEM images measuring the strain fields associated with each dislocation. These strain fields will affect the electrical and electronic properties of semiconductors by distorting the local bonding character. Charge transport and recombination may be altered through this effect on the electrostatic potential and via associated Cottrell atmospheres^35^. GPA is a method for obtaining local phase, which can be directly related to displacement field distorting the lattice fringes in the HRTEM or HAADF-STEM images with respect to the reference lattice^36^. Figure 4a–c presents the strain tensor component *ε*_*xx*_ of Type I, II, and III dislocations, respectively, and Fig. 4d present the εyy of Type III dislocation.

The strain fields of Lomer dislocations Type I and II extend over 3 nm radius showing the large region of influence around these cores. Type I and II dislocations show two compression-tension strain field pairs, in Fig. 4a,b. We note that GPA is not well suited to infer strain on the scale smaller than a unit cell and thus the fine features are likely an artifact. The long-ranged strain is real and we are particularly interested in its extent.

Figure 4c,d shows the strain tensor component ε_*xx*_ and ε_*yy*_ based on the HAADF-STEM image of the Type III dislocation obtained from the same area as shown in Fig. 3a. There is no in- plane strain expected due to the stacking fault itself. GPA shows very localized strain region around the stacking fault, which is again due to the small scale and thus can be ignored. The 30° Shockley partial dislocation has a very compact core and does not produce noticeable strain in this projection, although its screw component would need to be considered separately.

The lower (*a*/6)[141] core strain field is clearly much wider than that of the upper 30° partial dislocation, but appears less extended than the strain field of Type I and II cores. Strain fields from adjacent dislocation cores along the bicrystal interface are very close to overlapping with adjacent fields, but no actual interaction between adjacent strain fields has been observed, due to the average spacing between dislocation cores of ~5.6 nm. Thus, the individual cores can be considered as discrete and isolated from each other but the interface as a whole presents a continuously strained region.

### Electronic structure of CdTe dislocations

**Figure 5:**
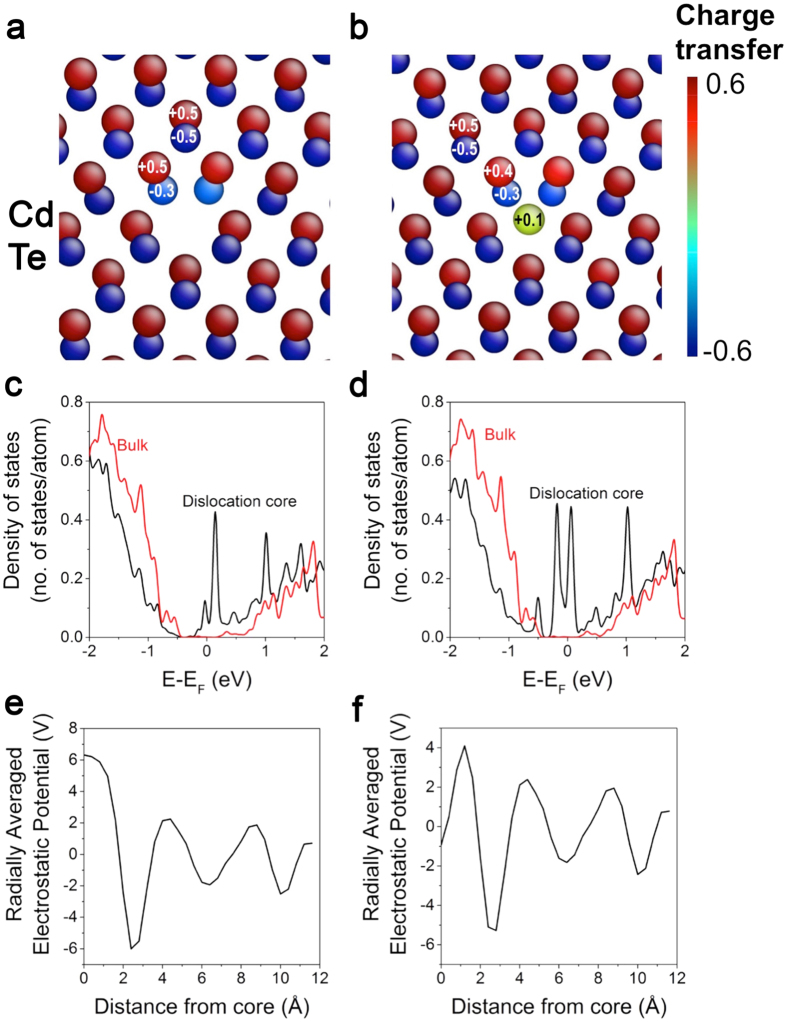
Electronic structure of Type I and II dislocation cores. Charge transfer around the dislocation cores calculated using Bader analysis is given in (**a,b**) for Type I and II dislocations, respectively. Radially integrated density of states (DOS) analysis is given in (**c**,**d**) for Type I and II, respectively. The DOS of a region far from the dislocation cores was included for a bulk reference. Radially averaged electrostatic potential profiles as a function of distance from the center of the dislocation cores for (**e**) Type I and (**f**) Type II.

First principles DFT calculations provide information about the local electronic structures of the dislocation cores. The amount of charge transfer near the Type I and II dislocation cores, calculated using Bader charge analysis, is given in Fig. 5a,b, respectively. In the Type I dislocation (Fig. 5a), the Cd (Te) atoms in the bulk (i.e. far from the core), have an average charge of +0.55 (−0.55) |*e*|, due to the polar-covalent character of the tetrahedral bonds between Cd and Te. In comparison, Te atoms surrounding the dislocation core have ~0.2 *|e|* smaller absolute Bader charges and the Cd atoms that form a dumbbell with these Te atoms have ~0.1 |*e*| smaller absolute Bader charges. The most significant effect is seen in the Type II dislocation (Fig. 5b), where the extra Cd atom at the center of the core has ~0.4 |*e*| lower absolute charge, becoming almost neutral in sharp contrast to the Cd atoms far from the dislocation core. The deviation of the Bader charges at the dislocation core compared to the bulk values is attributable to the change in coordination, which can give rise to the formation of additional states in the band gap.

Accordingly, the electronic structure of the dislocations is analyzed in terms of density of states (DOS). We integrate the projected DOS of atoms in a cylindrical volume around the dislocation core, and compare the result with the DOS of the bulk reference area, which is selected from the same computational cell far from the dislocation cores. Figure 5c,d shows the integrated DOS around Type I and II dislocation cores, respectively, near the Fermi level, compared to bulk reference in the simulation cell. Figure 5c,d clearly shows that the dislocation cores give rise to additional electronic states within the bulk band gap. While the bulk-like region in the calculation may not reproduce bulk CdTe exactly due to the residual strain field created by the dislocation cores, the bulk-like region exhibits a Kohn-Sham gap similar to that of bulk CdTe (~0.6 eV). The bulk-like region also shows negligible DOS within a gap of about 1.3 eV, similar to bulk CdTe.

The overall negative charge at the Type I dislocation core and positive charge at the Type II dislocation core shown in Fig. 5a,b are expected to lead to electron repulsion and attraction, respectively. Indeed, the radial profiles of the electrostatic potential (Fig. 5e,f) show a repulsive potential for electrons (i.e. attractive for holes) at the Type I core and attractive potential for electrons (repulsive for holes) at the Type II core. We thus see an opposing mechanism to recombination via the mid-gap states provided by the presence of charged Type I and II dislocation cores which can be expected to help separate photo-generated electron-hole pairs.

**Figure 6:**
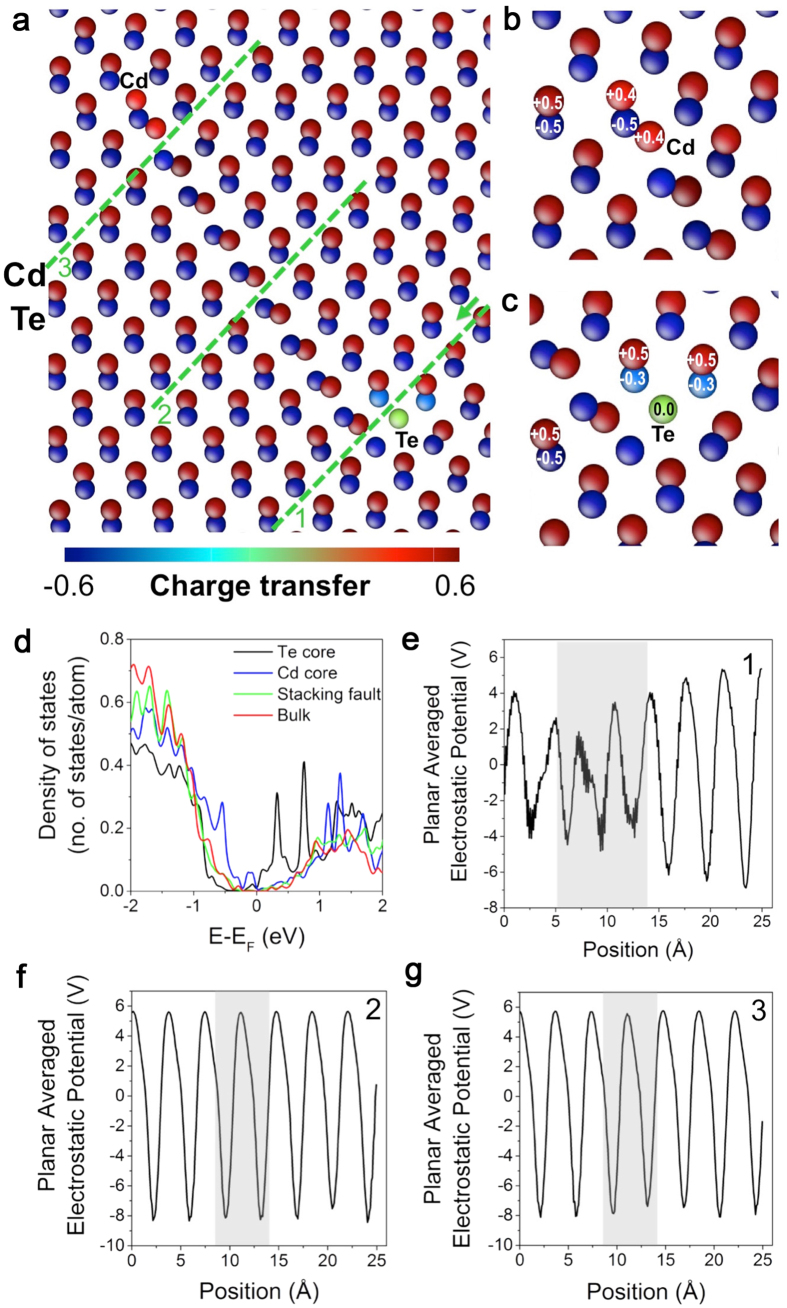
Electronic structure of Type III dislocation cores and stacking fault. (**a**) charge transfer distribution calculated using Bader charge analysis around the (**b**) Cd terminated and (**c**) Te-terminated dislocation cores, and stacking fault. (**d**) Radially integrated density of states (DOS) calculated around the Cd and Te terminated cores and at the middle of the stacking fault. The DOS of a region far from the stacking fault was included for a bulk reference. (**e**–**g**) Planar averaged electrostatic potential profiles computed along the lines 1–3, respectively as labeled in (**a**). The direction of the position is indicated with an arrow on line 1. In (**e**–**g**), shaded region corresponds to the location of dislocation core and stacking fault.

The amount of charge transfer near the Type III dislocation cores and stacking fault is shown in Fig. 6a, which indicate that the charge distribution around the stacking fault is the same as the bulk region away from the stacking fault. At the Cd-terminated dislocation core, as shown in Fig. 6b, the charge distribution is not significantly different compared to bulk region, with Cd atoms having only ~0.1 |e| smaller charge than the bulk. At the Te terminated core in Fig. 6c, the Te atoms surrounding the dislocation core have a charge of −0.3 |*e|*, which is ~0.2 |*e*| smaller in absolute value compared to the bulk region. Interestingly, the Te column at the dislocation core is charge neutral with almost zero charge (+0.04 |*e|*). The pertinent changes in the local atomic environment at the dislocation core will affect the electronic structure of the CdTe. In Fig. 6d, we calculate the integrated projected DOS of atoms in cylindrical regions centered at the dislocation cores at each end of the stacking fault, and in a rectangular region in the middle of the stacking fault, and compared the result with the DOS of the bulk reference area, which is selected from the same computational cell far from the stacking fault. The actual regions, where the DOS integration is carried out, is given in Supplementary Fig. 6. Figure 6d shows that stacking fault and bulk-like regions have similar DOS around the Fermi level, indicating that stacking fault does not alter the electronic structure of CdTe significantly. The Te-terminated core, on the other hand, shows formation of additional states in the midgap region up to the conduction band minimum. The Cd-terminated core shows additional states at the band gap that are closer to the valence band maximum.

The different charge states at the Te and Cd terminated cores at each end of the stacking fault are expected to act differently on charge carriers. To estimate the response of these defects on charge carriers, we computed the planar-averaged electrostatic potential along a direction perpendicular to the stacking fault. We considered different locations that are labeled in Fig. 6a, but used the same direction for averaging the electrostatic potential, as shown in Supplementary Fig. 7. Figure 6e shows the electrostatic potential profile along line 1, containing the Te-terminated dislocation core, which exhibits a lower electrostatic potential around the Te-core. The electrostatic potential profile along line 2, which passes through the middle of the stacking fault, indicates that the stacking fault exhibits bulk-like electrostatic potential profile (Fig. 6f). Similarly, electrostatic potential along line 3, containing Cd-terminated core, also appears bulk-like. Consequently, stacking fault and the Cd-terminated core is expected to have insignificant effects on charge separation and photovoltaic efficiency, similar to intrinsic twin boundaries^37^.

## Discussion

Grain boundaries, dislocations, and point defects in CdTe have attracted continuous interests due to the fact that the carrier recombination at these defects affects the CdTe solar cell performance. Most of earlier studies focused on Σ3 (111), and Σ9 (112) GBs. Dislocation cores in the 4.8° tilt (110) model grain boundary of CdTe, created using bicrystal fabrication, are studied in this work. Three types of Lomer dislocations are observed in the [1–10]/(110) 4.8° tilt grain boundary, all of which exhibit extended strain fields and midgap states that are expected to increase electron-hole pair recombination rates, and thereby decrease the performance of the solar-cell device. The proportion of Type I and Type II dislocations is more than 90%, since they are more stable than Type III dislocation according to Frank’s b^2^ criteria, as we discussed above. Type II dislocation needs a dangling bond, which means that it needs more energy to break the Cd-Te dumbbell structure, so the proportion of Type I is more than that of Type II.

**Figure 7:**
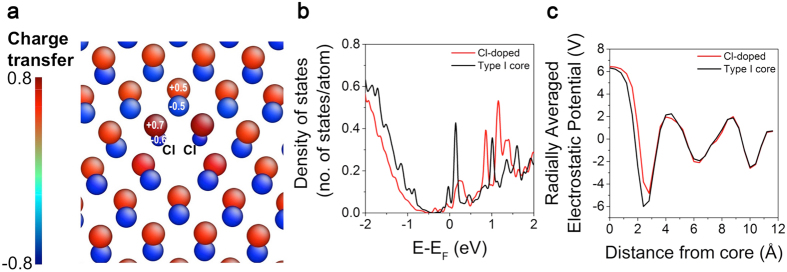
Electronic structure of Cl-doped Type I dislocation core. Charge transfer around the dislocation cores calculated using Bader analysis is given in (**a**). Radially integrated density of states (DOS) analysis is given in (**b**). The DOS of original Type I core is included for comparison. Radially averaged electrostatic potential profiles of Cl-doped and original Type I dislocation core as a function of distance from the center of the dislocation cores are given in (**c**).

Depending on whether the core has Cd or Te dangling bonds, Type I, II and III dislocation cores significantly changed the electronic structure of CdTe pertaining to photovoltaic properties, as shown in Figs 5 and 6. Here, we explore passivating the dangling bonds at the dislocation core by Cl atoms with the aim of understanding its role on improving photovoltaic efficiency of CdTe that was reported recently^38^,^39^. As Cl impurities prefer to segregate at dislocation cores and grain boundaries^38^, we substitute all of the Te atoms at the Type I core with Cl to observe the maximum effect of the changes in the electronic structure and fully relax the atomic positions. Figure 7a shows the relaxed structure of the Cl-doped Type I dislocation core and Bader charge distribution. Cl atoms had −0.6 |*e|* charge and the nearest Cd atoms had a charge of +0.7 |*e|*. The absolute charge values on Cd and Cl at the core are larger than the Type I core in Fig. 5a. The density of states of Cl-doped core compared to original Type I core is given in Fig. 7b, showing substantial reduction in the electronic states in the gap due to Cl doping, which indicates that Cl is effectively passivating the dangling bonds at the dislocation core. However, the amount of Cl considered here was not sufficient to completely eliminate the additional states at the midgap. On the other hand, the radially averaged electrostatic potential of the Cl-doped core does not show a significant change compared to the original Type I core as shown in Fig. 7c, such that Cl-doped core still had a negative charge and would expect to attract holes and facilitate charge separation. Therefore, Cl doping of dislocation cores reduces the midgap states but in the mean time maintains charge separation that would effectively be expected to increase the lifetimes and hence, the photovoltaic efficiency of CdTe.

In a previous study^5^, single Cd and Te columns have been found at the CdTe intragrain Shockley partial dislocation cores, where the stacking faults terminate. The electronic structure of the individual dislocation cores reveals that the Cd cores act as shallow donors while the Te cores act as shallow acceptors^5^. While each individual core modeled separately may appear to have benign characteristics, there is unmistakable charge transfer between those cores when they appear at the ends of the same stacking fault, which is consistent with our results. Moreover, our models, which are not idealized models of separated cores, but realistic models consisting of up to ~1900 atoms and shown to match the STEM images, showed that more generally, Type I, II and III dislocation cores are not electrically benign, as the Cd and Te terminating atoms at the dislocation cores are under-coordinated.

In summary, we create a [1–10]/(110) 4.8° tilt grain boundary via wafer bonding. This study reveals two types of Lomer (*a*/2)[110] undissociated dislocations with different atom configurations, and one kind of Lomer (*a*/2)[110] dislocation dissociated to an intrinsic stacking fault with a 30° Shockley partial and an unusual (*a*/6)[141] partial dislocations. We study the reaction mechanism combining the HAADF-STEM image, GPA strain maps, and Thomson tetrahedron. Structure models derived from the STEM images have been constructed and relaxed by DFT calculations. The relaxed structure is found to be in good agreement with the experiments images. DFT calculations show all three types dislocations present midgap states that are expected to increase recombination, but also electrostatic potential profiles that may aid charge separation. We find that Cl passivation at type I core reduces the midgap states without affecting charge separation. Further work will focus on electrical measurement to verify the solar cell performance on this kind of GB. The combination of STEM, GPA and DFT calculation to explore the atomic structure, dislocation formation, strain map and electronic structure of dislocation cores should be applicable to other complicated GBs in a wide range of II-VI and III-V semiconductors.

## Methods

### Bicrystal fabrication

High purity, one side polished, 5 × 10 mm^2^ *p*-type CdTe (111) wafers with a thickness of 3 mm were used for the bonding experiments. Out-of-plane (2*θ*/*ω* scan) and in-plane (*φ* scan) X-ray diffraction (XRD) (Ultima III, Rigaku) was used to determine the crystal orientations of the planes and flats, respectively. The root mean square surface roughness (RMS) was measured by atomic force microscopy (AFM) (Dimension 3100, Veeco) in tapping mode. For the cleaning process, samples were rinsed in de-ionized water for 5 s, briefly dipped in hydrochloric acid (15% HCl) for 30 s and then rinsed in de-ionized water for 5 s. The samples were dried under a flow of nitrogen gas, and heated on a hot plate at 110 °C for 5 s to evaporate the de-ionized water absorbed on the surface. After that, the wafer pair was loaded into a vacuum bonder (EV 501, EV Group) immediately, to prevent oxidation of the CdTe surface. Bonding was performed at 400 °C for 20 hrs under a pressure of 1 MPa.

### TEM imaging conditions and GPA

Local structure and diffraction patterns were acquired using HRTEM (2100F, JEOL) of Focused Ion Beam (FIB) (Nova 200, FEI) milled cross-sections. The atomic arrangement of the interface was characterized using HAADF-STEM (JEM-ARM200F, JEOL). Geometrical phase analysis (GPA) was performed based on the HAADF-STEM images using a GPA plug-in package, which was implemented in the Digital Micrograph software (Gatan).

### Construction of dislocation core atomic structures using image analysis and empirical potentials

Atomistic models of pairs (dipoles) of dislocation cores are constructed from HAADF-STEM images and empirical potentials. First, a black and white filter was applied on the raw HAADF-STEM image to remove the noise and enhance the bright spots located at atomic columns. The positions of bright spots that correspond to atomic columns were identified using image analysis techniques with the Scikit-Image package. To extract the coordinates of atomic columns corresponding to bright spots, we located the maximum peak intensities using a maximum filter with a determined threshold on the image. To remove multiple maximum peaks appearing between atomic columns, we calculated the nearest neighbors of each peak using a radius of 0.9 Å, and combined the peak locations that are closer than this value by taking the center of mass of the nearest neighbors of each peak. After locating the positions of atomic columns, we used the crystallographic information in the CdTe crystal to identify the separate Cd and Te columns and obtained the atomic structure. To build a periodic supercell, we juxtaposed the structure with its mirror image and created a dislocation dipole where necessary. Before first principles calculations, we relaxed the dislocation dipole structure using available Stillinger-Weber^40^ and bond order empirical potentials^41^ for CdTe. These calculations have lower computational costs compared to DFT calculations and enabled us to determine near-minimum-energy dislocation core structures which match well with the HAADF-STEM images. We considered many variations of atomic configuration at the dislocation cores by adding/subtracting atoms and changing the dimensions of the supercell so that the dislocation cores are not strongly affected by the periodic boundary conditions imposed on the system. In all cases, both interatomic potentials relaxed the atoms to similar positions. The atomic coordinates in the dislocation models are further relaxed using DFT.

The Type I dislocation dipole cell consisted of 1440 atoms with 720 Cd and 720 Te. Type II differs from Type I by only a single Cd/Te atom at the dislocation dipole cores. Type III consists of a stacking fault with two dislocation cores at the two ends. The Cd-terminated upper core structure was apparent from STEM image, but there is ambiguity in the lower core atomic structure. Initially, we created a supercell of 56 × 57 × 15 Å containing 1401 atoms (697 Cd and 704 Te), and attempted different variations of single Te, single Cd, Cd-Te dumbbell atomic columns at the lower core. We compared the relaxed structures with the STEM image and found that this size was insufficient for overcoming the periodic boundary effects, so that we increased the cell size to 70 × 64 × 15 Å containing 1917 atoms (957 Cd and 960 Te), which is sufficient for reducing strain imposed on atoms around the dislocation cores. We tested similar atomic and elemental variations at the lower dislocation core using this large simulation cell as well. Finally, we chose the structure with Te-termination at the lower core that had the best match with STEM around the dislocation core and stacking fault in order to determine the electronic properties.

### DFT calculations

The electronic structure of dislocation cores was analyzed in terms of electronic density of states, charge transfer, and electrostatic potential change at and near the dislocation core using planewave-based density functional theory (DFT) method. All DFT calculations were carried out using Vienna Ab initio Simulation Package (VASP)^42^,^43^. Projector augmented wave (PAW) potentials^44^ were used, and the exchange-correlation was treated with the generalized gradient approximation (GGA) parameterized by Perdew, Burke, and Ernzerhof (PBE)^45^. Calculations were carried out at the Γ-point in the Brilluoin zone using planewave kinetic energy cutoff of 343 eV. Atomic positions were relaxed until forces are below 0.05 eV/Å and electronic relaxation was converged to 10^−5^ eV to ensure convergence to 1–2 meV/atom in total energy. The electronic charges on atoms in DFT calculations are obtained by partitioning the charge density according to atomic positions in the crystal based on the Bader charge analysis^46^, as implemented in VTST-scripts^47^. Some DFT calculations are performed on Extreme Science and Engineering Discovery Environment (XSEDE) resources^48^.

## Additional Information

**How to cite this article**: Sun, C. *et al.* Atomic and electronic structure of Lomer dislocations at CdTe bicrystal interface. *Sci. Rep.* **6**, 27009; doi: 10.1038/srep27009 (2016).

## Supplementary Material

Supplementary Information

## References

[b1] Zweibel K. The impact of tellurium supply on cadmium telluride photovoltaics. Science 328, 699–701 (2010). 20448173 10.1126/science.1189690

[b2] Green M. A., Emery K., Hishikawa Y., Warta W. & Dunlop E. D. Solar cell efficiency tables (Version 45). Prog. Photovolt: Res. Appl. 23, 1–9 (2015).

[b3] Shockley W. & Queisser H. J. Detailed Balance Limit of Efficiency of *p‐n* Junction Solar Cells. J. Appl. Phys. 32, 510–519 (1961).

[b4] Green M. A. Radiative efficiency of state-of-the-art photovoltaic cells. Prog. Photovolt: Res. Appl. 20, 472–476 (2011).

[b5] Li C. *et al.* Carrier separation at dislocation pairs in CdTe. Phys. Rev. Lett. 111, 096403 (2013). 24033055 10.1103/PhysRevLett.111.096403

[b6] Metzger W. K. & Gloeckler M. The impact of charged grain boundaries on thin-film solar cells and characterization. J. Appl. Phys. 98, 063701 (2005).

[b7] Duenow J. N. *et al.* Single-crystal CdTe solar cells with Voc greater than 900 mV. Appl. Phys. Lett. 105, 053903 (2014).

[b8] Zhang L. *et al.* Effect of copassivation of Cl and Cu on CdTe grain boundaries. Phys. Rev. Lett. 101, 155501 (2008). 18999610 10.1103/PhysRevLett.101.155501

[b9] Lymperakis L., Neugebauer J., Albrecht M., Remmele T. & Strunk H. Strain induced deep electronic states around threading dislocations in GaN. Phys. Rev. Lett. 93, 196401 (2004). 15600857 10.1103/PhysRevLett.93.196401

[b10] Blavette D., Cadel E., Fraczkiewicz A. & Menand A. Three-dimensional atomic-scale imaging of impurity segregation to line defects. Science 286, 2317–2319 (1999). 10600736 10.1126/science.286.5448.2317

[b11] Wang Z., Saito M., McKenna K. P. & Ikuhara Y. Polymorphism of dislocation core structures at the atomic scale. Nat. Commun. 5, 3239 (2014). 24476810 10.1038/ncomms4239

[b12] Paulose J., Chen B. G.-G. & Vitelli V. Topological modes bound to dislocations in mechanical metamaterials. Nature Phys. 11, 153–156 (2015).

[b13] Nakamura A., Matsunaga K., Tohma J., Yamamoto T. & Ikuhara Y. Conducting nanowires in insulating ceramics. Nature Mater. 2, 453–456 (2003). 12806386 10.1038/nmat920

[b14] Ikuhara Y. Nanowire design by dislocation technology. Prog. Mater. Sci 54, 770–791 (2009).

[b15] Duscher G., Chisholm M. F., Alber U. & Rühle M. Bismuth-induced embrittlement of copper grain boundaries. Nature Mater. 3, 621–626 (2004). 15322533 10.1038/nmat1191

[b16] Takehara K., Sato Y., Tohei T., Shibata N. & Ikuhara Y. Titanium enrichment and strontium depletion near edge dislocation in strontium titanate [001]/(110) low-angle tilt grain boundary. J. Mater. Sci. 49, 3962–3969 (2014).

[b17] Li C. *et al.* From atomic structure to photovoltaic properties in CdTe solar cells. Ultramicroscopy 134, 113–125 (2013).

[b18] Zhou X., Ward D. K., Wong B. M., Doty F. P. & Zimmerman J. A. Molecular dynamics studies of dislocations in CdTe crystals from a new bond order potential. J. Phys. Chem. C 116, 17563–17571 (2012). 10.1021/jp3039626 PMC3433129 22962626

[b19] Paulauskas T. *et al.* Atomic scale study of polar Lomer–Cottrell and Hirth lock dislocation cores in CdTe. Acta Cryst. A 70, 524–531 (2014).

[b20] Zhou X. W., Ward D. K., Wong B. M. & Doty F. P. Melt-growth dynamics in CdTe crystals. Phys. Rev. Lett. 108, 245503 (2012). 23004290 10.1103/PhysRevLett.108.245503

[b21] Yan Y., Jones K. M., Al-Jassim M. M., Dhere R. & Wu X. Transmission electron microscopy study of dislocations and interfaces in CdTe solar cells. Thin Solid Films 519, 7168–7172 (2011).

[b22] Lu P. & Smith D. J. Dissociated 60° dislocations in CdTe studied by high-resolution electron microscopy. Philos. Mag. B 62, 435–450 (1990).

[b23] Sun C. *et al.* Creating a single twin boundary between two CdTe (111) wafers with controlled rotation angle by wafer bonding. Appl. Phys. Lett. 103, 252104 (2013).

[b24] Plößl A. & Kräuter G. Wafer direct bonding: tailoring adhesion between brittle materials. Mater. Sci. Eng. R 25, 1–88 (1999).

[b25] Kim M. J. & Carpenter R. W. Heterogeneous silicon integration by ultra-high vacuum wafer bonding. J. Electron. Mater. 32, 849–854 (2003).

[b26] Kim M. J., Carpenter R. W., Cox M. J. & Xu J. Controlled planar interface synthesis by ultrahigh vacuum diffusion bonding/deposition. J. Mater. Res. 15, 1008–1016 (2000).

[b27] Medlin D. L., Erickson K. J., Limmer S. J., Yelton W. G. & Siegal M. P. Dissociated 1/3<111> dislocations in Bi2Te3 and their relationship to seven-layer Bi3Te4 defects. J. Mater. Sci. 49, 3970–3979 (2014).

[b28] Tochigi E., Shibata N., Nakamura A., Yamamoto T. & Ikuhara Y. Partial dislocation configurations in a low-angle boundary in α-Al_2_O_3_. Acta Mater. 56, 2015–2021 (2008).

[b29] Heinemann S., Wirth R., Gottschalk M. & Dresen G. Synthetic [100] tilt grain boundaries in forsterite: 9.9 to 21.5°. Phys. Chem. Minerals 32, 229–240 (2005).

[b30] Hornstra J. Dislocations in the diamond lattice. J. Phys. Chem. Solids 5, 129–141 (1958).

[b31] Nandedkar A. S. & Narayan J. Atomic structure of dislocations and dipoles in silicon. Philos. Mag. A 56, 625–639 (2006).

[b32] Geipel T., Xiao S. Q. & Pirouz P. HRTEM study of 1/6<411> partial dislocations in hot hardness indented Ge. Philos. Mag. Lett. 67, 245–251 (1993).

[b33] Grilhé J., Seshan K. & Washburn J. On the possibility of nucleating loops with burgers vector (DC′) by the clustering of interstitials. Radiation Effects 27, 115–118 (2006).

[b34] Hirth J. P. & Lothe J. The theory of dislocations. (McGraw Hill, 1968).

[b35] Alexander H. & Teichler H. Handbook of Semiconductor Technology (Wiley-VCH, Berlin, 2000).

[b36] Hÿtch M. J., Snoeck E. & Kilaas R. Quantitative measurement of displacement and strain fields from HREM micrographs. Ultramicroscopy 74, 131–146 (1998).

[b37] Buurma C., Paulauskas T., Guo Z., Klie R. & Chan M. K. Y. Density Functional Theory Modeling of Twin Boundaries in CdTe as Informed by STEM Observations. Microsc. Microanal. 20, 528–529 (2014).

[b38] Li C. *et al.* Grain-Boundary-Enhanced Carrier Collection in CdTe Solar Cells. Phys. Rev. Lett. 112, 156103 (2014). 24785058 10.1103/PhysRevLett.112.156103

[b39] Metzger W. K., Albin D., Romero M. J., Dippo P. & Young M. CdCl_2_ treatment, S diffusion, and recombination in polycrystalline CdTe. J. Appl. Phys. 99, 103703 (2006).

[b40] Wang Z. Q., Stroud D. & Markworth A. J. Monte- Carlo study of the liquid CdTe surface. Phys. Rev. B 40, 3129–3132 (1989). 10.1103/physrevb.40.3129 9992248

[b41] Ward D. K., Zhou X. W., Wong B. M., Doty F. P. & Zimmerman J. A. Analytical bond-order potential for the cadmium telluride binary system. Phys. Rev. B 85, 115206 (2012).

[b42] Kresse G. & Furthmüller J. Efficient iterative schemes for ab initio total-energy calculations using a plane-wave basis set. Phys. Rev. B 54, 11169–11186 (1996). 10.1103/physrevb.54.11169 9984901

[b43] Kresse G., Kresse G. & Furthmüller J. Efficiency of ab-initio total energy calculations for metals and semiconductors using a plane-wave basis set. Comput. Mater. Sci. 6, 15–50 (1996).

[b44] Kresse G. & Joubert D. From ultrasoft pseudopotentials to the projector augmented-wave method. Phys. Rev. B 59, 1758–1775 (1999).

[b45] Perdew J. P., Burke K. & Ernzerhof M. Generalized gradient approximation made simple. Phys. Rev. Lett. 77, 3865–3868 (1996). 10062328 10.1103/PhysRevLett.77.3865

[b46] Bader R. F. W. Atoms in molecules. Acc. Chem. Res. 18, 9–15 (1985).

[b47] Henkelman G., Arnaldsson A. & Jonsson H. A fast and robust algorithm for Bader decomposition of charge density. Comput. Mater. Sci. 36, 354–360 (2006).

[b48] Towns J. *et al.* XSEDE: Accelerating scientific discovery. Comput. Sci. Eng. 16, 62–74 (2014).

