# Supplementary material for: Atomic and electronic structure of Lomer dislocations at CdTe bicrystal interface
Source: Sci Rep. 2016 Jun 3;6:27009. doi: 10.1038/srep27009 (PMC4891715; doi:10.1038/srep27009)
Supplement: Supplementary Information [file srep27009-s1.pdf]

Supplementary Information

Atomic and electronic structure of Lomer dislocations at CdTe bicrystal interface

Ce Sun[1,*], Tadas Paulauskas[2,*], Fatih G. Sen[3,*], Guoda Lian[1], Jinguo Wang[1], Christopher Buurma[2], Maria K. Y. Chan[3], Robert F. Klie[2] & Moon J. Kim[1]

[1]Department of Materials Science and Engineering, the University of Texas at Dallas, Richardson, TX 75080, USA.

[2]Department of Physics, University of Illinois at Chicago, Chicago, IL 60607, USA.

[3]Center for Nanoscale Materials, Argonne National Laboratory, Lemont, IL 60439, USA.

[*]These authors contributed equally to this work. Correspondence and requests for materials should be addressed to M.J.K. (email: moonkim@utdallas.edu).

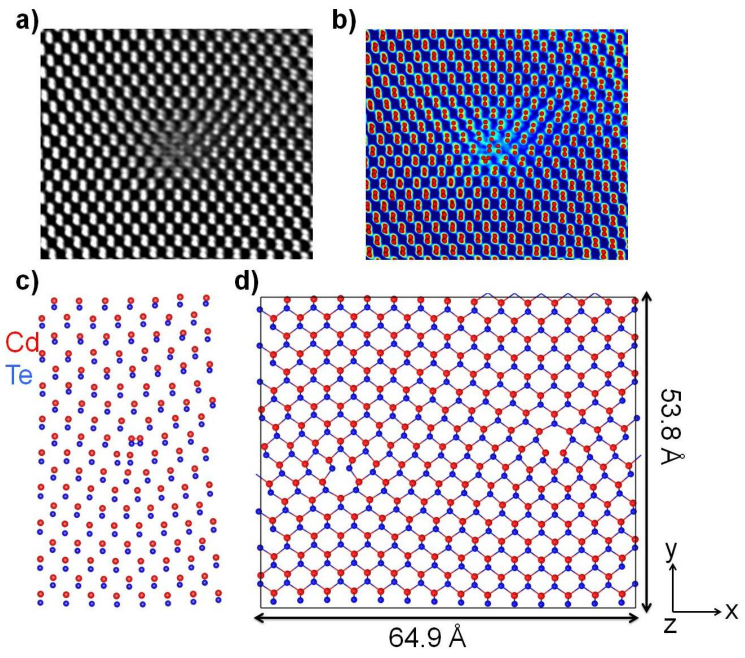

Supplementary Figure 1 | Construction of the atomic structure of dislocation cores from STEM image using image analysis. STEM image of dislocation cores are processed with image analysis methods using the Scikit-Image package to construct two-dimensional atomic positions. (**a**) Black and white filtered image of dislocation core type I. (**b**) Coordinates of maximum peak intensities using maximum filter with a threshold of 0.03 on the image as indicated by red dots from (a). (**c**) Constructed atomic structure for dislocation type I. (d) Periodic cell constructed by combining the mirror image of the atomic structure in (a), which was relaxed with the Stillinger-Weber potential. We added two additional layers in the in the z-direction to reduces interactions between periodic images.

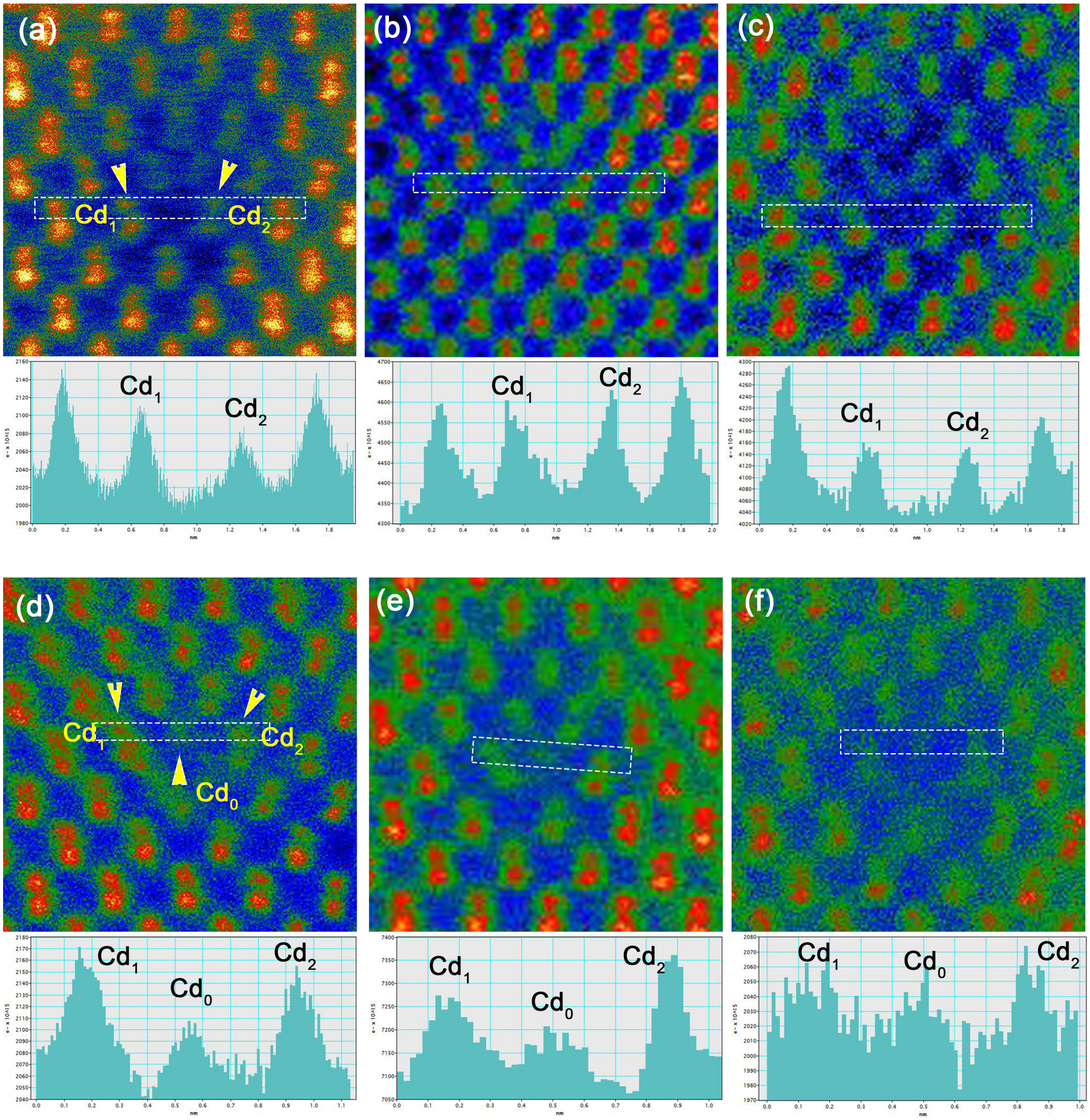

Supplementary Figure 2 | Atomic armaments and line profiles of dislocations. (**a**)-(**c**) STEM images and line profile of Type I dislocations, the profiles showed the distance between Cd_1 and Cd_2 is about 0.65 nm. (**d**)-(**e**) STEM images and line profile of Type II dislocations, the profiles showed an extra column (Cd_0) between Cd_1 and Cd_2, and the distance the distance between Cd_1 and Cd_2 increased to about 0.72 nm.

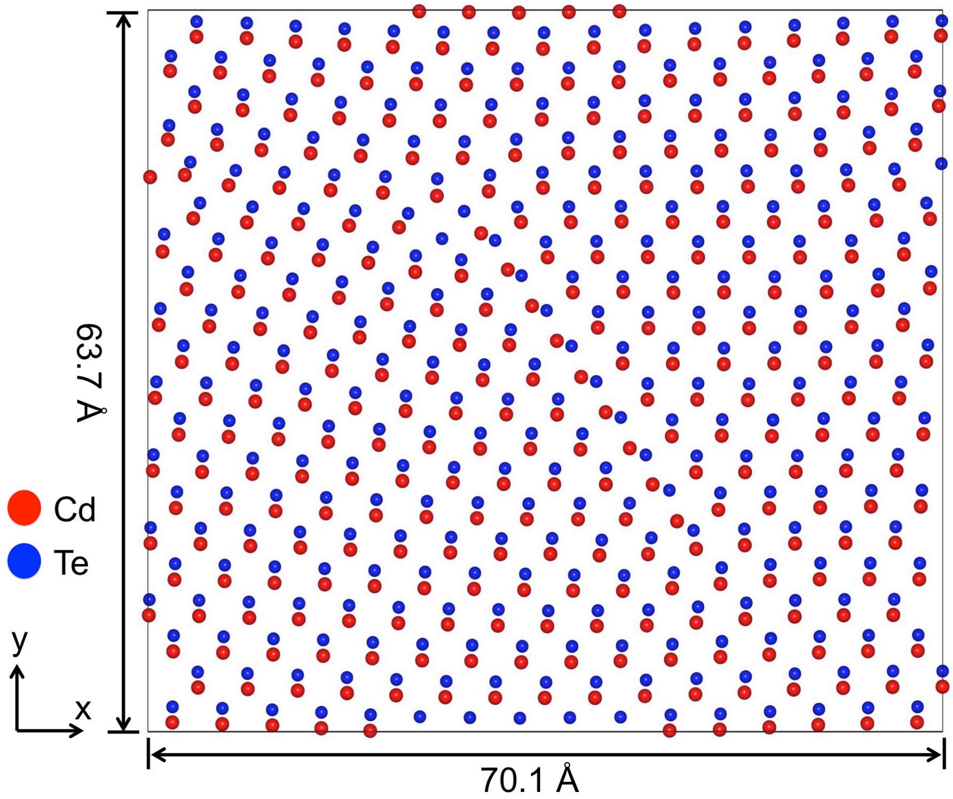

Supplementary Figure 3 | Atomic structure of Type III dislocation cores and stacking fault.
The unit cell has dimension of 70.1 × 63.7 × 15.3 Å consisting of 1917 atoms.

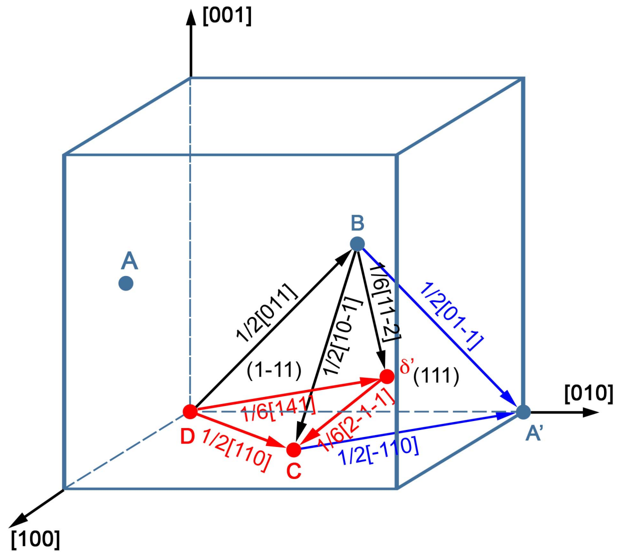

Supplementary Figure 4 | Modified Thompson tetrahedron. Thompson tetrahedron has been used to study the reaction dislocations. The reaction of Type I and II Lomer dislocations can be interpreted as two merged 60° elemental dislocations (*a*/2)[011] + (*a*/2)[10-1] → (*a*/2)[110] (DB + BC → DC). In order to understand how Type III dislocation formed, we may consider two probable scenarios: i) the dissociation of a previously perfect Lomer dislocation or ii) the reaction of a 60° dislocation with an already dissociated 60° dislocation. Frank's $\mathbf{b}^2$ criteria, which compares elastic energy states of dislocations prior and post reaction, tells us that it is energetically more favorable for the latter process to take place. In fact, it can be seen that the undissociated Lomer dislocation has lower elastic energy compared to the final product after dissociation into two partials and a stacking fault. However, the elastic energy is reduced when two isolated dislocations, one of which is already dissociated, merge. In the following we will assume the latter scenario and discuss the reaction in more details. The dissociation of a 60° dislocation on the (111) plane can be written as: (*a*/2)[10-1] (60°) → (a/6)[2-1-1] (30°) + (a/6)[11-2] (90°) or using the Thomson tetrahedron notation, as: BC(60°) → δ'C (30°) + Bδ' (90°). An intrinsic stacking fault is created in this step and the 30° partial is placed at the upper

core of Fig. 3a. The second 60° dislocation reacts with the 90° partial (*a*/6)[11-2] or Bδ' in Supplementary Fig. 3 to form the partial dislocation at the lower core of Fig 3c, described as: (*a*/2)[011] (60°) + (*a*/6)[11-2] (90°) → (*a*/6)[141]. The dislocation reactions of the whole Type III dislocation therefore comes out as: (*a*/2)[10-1] + (*a*/2)[011] → (a/6)[2-1-1] + (a/6)[11-2] + (*a*/2)[011] → (a/6)[2-1-1] + (*a*/6)[141].

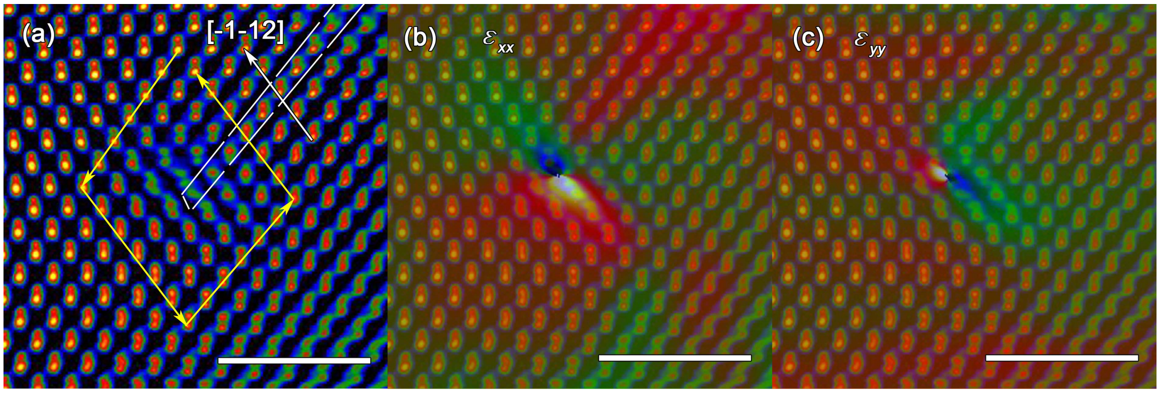

Supplementary Figure 5 | 60° dislocation core. (**a**) A 60° dislocation at the bonded interface with a extra plane marked by a white box. The Burgers circuit marked by yellow arrows indicates the projection of the Burgers vector is (*a*/4)[-1-12], which is the projected component of the Burgers vector for a 60° dislocation in the current [1-10] projection. Scale bar, 2 nm. (**b**) and (**c**) Strain components of ε_{xx} and ε_{yy} for the 60° dislocation structure. The HAADF-STEM images are overlapped on the strain maps to indicate the exact location of the dislocation core, and the only one compression-tense strain field pair. Scale bar, 2 nm.

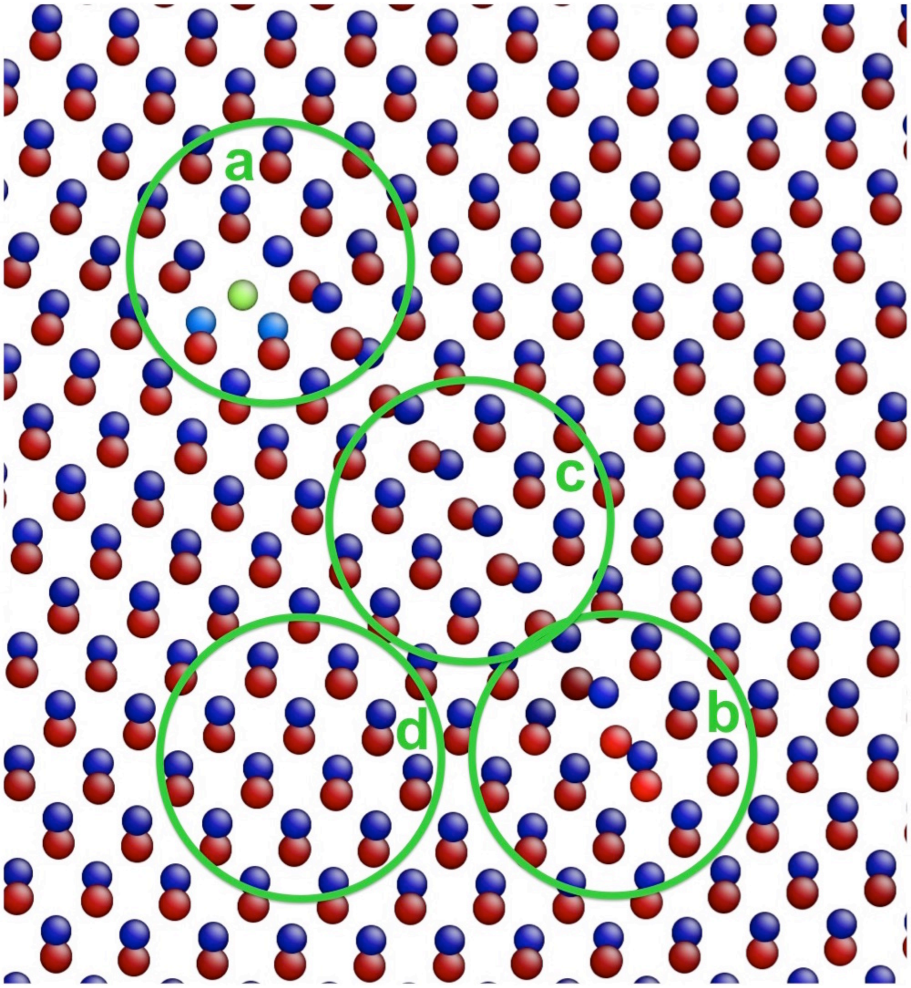

Supplementary Figure 6 | Locations for radially integrated total density of states (DOS). (a) Te core, (b) Cd core, (c) stacking fault, and (d) bulk reference.

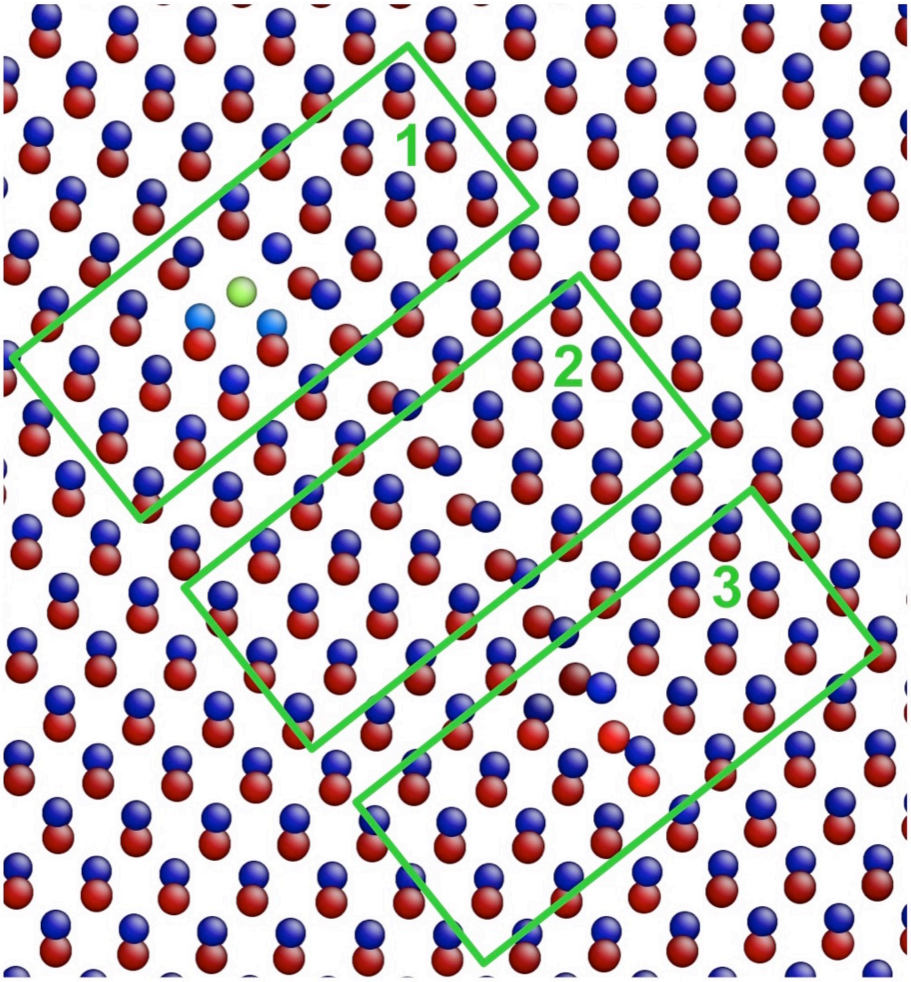

Supplementary Figure 7 | Locations for planar averaged electrostatic potentials. Planar averaging carried out along a direction perpendicular to the stacking fault line. Total length of each line was 25 Å and the width of each region is 15 Å.
